# A Continuation Technique for Maximum Likelihood Estimators in Biological Models

**DOI:** 10.1007/s11538-023-01200-0

**Published:** 2023-08-31

**Authors:** Tyler Cassidy

**Affiliations:** School of Mathematics, University of Leeds, Leeds, LS2 9JT UK

**Keywords:** Parameter estimation, Numerical continuation, Model calibration, Experimental design

## Abstract

Estimating model parameters is a crucial step in mathematical modelling and typically involves minimizing the disagreement between model predictions and experimental data. This calibration data can change throughout a study, particularly if modelling is performed simultaneously with the calibration experiments, or during an on-going public health crisis as in the case of the COVID-19 pandemic. Consequently, the optimal parameter set, or maximal likelihood estimator (MLE), is a function of the experimental data set. Here, we develop a numerical technique to predict the evolution of the MLE as a function of the experimental data. We show that, when considering perturbations from an initial data set, our approach is significantly more computationally efficient that re-fitting model parameters while producing acceptable model fits to the updated data. We use the continuation technique to develop an explicit functional relationship between fit model parameters and experimental data that can be used to measure the sensitivity of the MLE to experimental data. We then leverage this technique to select between model fits with similar information criteria, a priori determine the experimental measurements to which the MLE is most sensitive, and suggest additional experiment measurements that can resolve parameter uncertainty.

## Introduction

As quantitative modeling becomes more prevalent across biology and medicine (Altrock et al. 2015; Perelson 2002; Sanche et al. 2020), mathematical models are increasingly being developed during the experimental data collection that will inform model parameters. This cooperation facilitates the use of mathematical modelling to inform experimental design and suggest potential intervention strategies (Zhang et al. 2022; Sanche et al. 2020; Cárdenas et al. 2022; Luo et al. 2022). The COVID-19 pandemic is a striking example of the resulting feedback loop, where mathematical models suggest intervention strategies that influence the evolving public health crisis before being re-calibrated to new data. (Holmdahl and Buckee 2020; Thompson 2020; Davies et al. 2020).

Each updated data set requires re-calibration of the model typically through computationally expensive optimization techniques. To reduce this computational cost of the re-calibration step, it is common to use the existing parameters as a starting point when performing parameter fitting to incoming experimental data sets. This approach recycles optimization work but does not utilize leverage the relationship between the initial and updated experimental data set. Here, we present a computational method to incorporate information about evolving data sets during the model validation and parameter estimation steps.

Specifically, for given model parameters and an initial experimental data set, we develop a method to predict the best-fit parameter set to an updated experimental data set. Our approach can be viewed as a numerical continuation technique (Dhooge et al. 2008; De Souza and Humphries 2019). However, rather than studying the dynamical properties of the mathematical model as a function of model parameters, we consider the evolution of best-fit model parameters as a function of the experimental data. We use the necessary condition for a local optima to write the best-fit parameters as an implicit function of the experimental data. We can then predict best-fit parameter sets for evolving experimental data without performing any optimization. Avoiding optimization leads to significant computational savings and we demonstrate these gains via two examples. In both these examples, our prediction method produces comparable model fits to randomly perturbed data sets as optimization techniques without the computational cost of solving the inverse optimization problem.

While our approach does lead to increased computational efficiency, the more immediate application of our work may be in experimental design. Specifically, we identify an explicit relationship between individual best-fit parameter values and individual experimental data points through our continuation approach. We can therefore quantify which experimental measurements are the most informative for determining best-fit parameters and measure the sensitivity of parameter estimates to perturbations in data. The role of experimental design in model selection and parameterization has been extensively studied (Silk et al. 2014; Cárdenas et al. 2022; Li and Vu 2015, 2013). In particular, Li and Vu (2015) studied how correlations between best-fit model parameters can impact practical and structural identifiability of model parameters while Silk et al. (2014) and Cárdenas et al. (2022) explored how experimental design impacts model selection from a class of possible mathematical models. Conversely, our contribution explicitly relates individual experimental measurements with individual best-fit parameter estimates. We explicitly link our continuation technique to the Fisher information matrix commonly used in optimal experimental design (Kreutz and Timmer 2009; Braniff et al. 2019). Taken together, our approach allows the increased confidence in model parametrization from optimal experimental design to be mapped directly to individual model parameters. Accordingly, we can therefore design experiments to address specific uncertainties in parameter estimates.

Furthermore, our work offers a distinct step towards understanding how robust parameter estimates are to evolving data. Many existing computational methods quantify confidence in parameterization; formal parameter sensitivity analyses (Marino et al. 2008; Maiwald et al. 2016; Zi 2011), virtual population approaches (Allen et al. 2016; Cassidy and Craig 2019; Jenner et al. 2021), or parameter identifiability analysis (Castro and de Boer 2020), often via profile likelihood computation (Raue et al. 2009, 2014; Kreutz et al. 2012), quantify how robust model predictions are to parameter variation. In particular, these techniques view the experimental data as fixed up to experimental noise and focus on the relationship between model parameters and model predictions. We offer a complementary approach to existing sensitivity analysis by explicitly studying how the best-fit parameters vary due to changes in calibration data. As we will see, our approach encodes information from local sensitivity analysis when calculating the functional relationship between the best-fit parameters and the calibration data. Consequently, while classical sensitivity analysis quantifies variability in model output due to change in model parameters, our approach considers changes in model parameters, and thus model predictions, as a function of the calibration data. We demonstrate this mapping of experimental data to best-fit parameter via an example drawn from mathematical oncology (Cassidy et al. 2021). These results, when combined with existing information criteria like the AIC or BIC (Kass and Raftery 1995), allow for modellers to quantify the robustness of best-fit parameter estimates when comparing different model fits to experimental data.

The remainder of the article is structured as follows. We begin by defining the optimization problem in Sect. 2.1. We develop the continuation method in Sect. 2.2, discuss our numerical implementation in 2.3, and explore the connection between our continuation approach and optimal experimental design in 3.1. We then turn to two examples from mathematical biology to illustrate the utility of our technique in Sect. 3.2 before finishing with a brief discussion.

## Methods

### Formulation of the Optimization Problem

Here, we introduce the framework of the underlying optimization problem. We focus on ordinary differential equation (ODE) models representing biological processes, as these models are common throughout mathematical biology. However, our approach extends to partial differential equation or delay differential equation models directly. We consider a generic ODE based model throughout the remainder of this work.

Let the model states be given by $x(t) \in \mathbb {R}^n$ with model parameters denoted by $\theta \in \Omega \subset \mathbb {R}^p$ where $\Omega$ is a subset of biologically plausible parameter values. We allow the initial condition *x*(0) to depend on the model parameters $\theta$. Taken together, we consider the differential equation model

1 $$\begin{aligned} \frac{\textrm{d}}{\textrm{dt}}x(t) = f(x,\theta ); \quad x(0) = x_0(\theta ) \end{aligned}$$

where *f* is continuously differentiable in *x* and $\theta$.

We consider calibration data $\{ \phi _i \}_{i=1}^{d \times m}$ representing *m* measurements each taken at *d* time points $\{ t_i \}_{i=1}^d$. It is possible that model species are not directly comparable against the calibration data so we define the *m* model observables by

$$\begin{aligned} y_i(\theta ) = h(x(t_i,\theta ),\theta ) \in \mathbb {R}^{d \times m}. \end{aligned}$$

In what follows, we consider $m=1$ for notational simplicity although the analysis extends for $m \geqslant 2$.

#### Likelihood function and objective function

##### Remark 1

The methods that follow do not assume a specific objective function. However, we do assume that the objective function is twice continuously differentiable as is commonly the case. For simplicity, we present the remainder of our results using the common log-likelihood formulation (Stapor et al. 2018; Maiwald et al. 2016).

The likelihood describes the probability of observing experimental data $\phi$ as a function of $\theta$ and is given by

2 $$\begin{aligned} \mathcal {L}(y(\theta ),\phi ) = \prod _{i=1}^d \frac{1}{\sqrt{ 2\pi \sigma ^2_{i} }} \exp \left[ -\frac{(y_i(\theta )- \phi ^*_i)^2}{\sigma _i^2} \right] \end{aligned}$$

The experimental error at each measurement point, $\sigma _i$, can be estimated as an additional model parameter or fixed to a known value. Here, we follow Sharp et al. (2022) and take $\sigma _i$ fixed at a known constant value, although it is possible to include $\sigma _i$ in the vector of unknown parameters $\theta$. The maximum likelihood estimator (MLE) $\theta ^*$, and thus best-fit model parameters for the given experimental data $\phi$, is defined by the solution of the inverse problem

3 $$\begin{aligned} \theta ^* = \textrm{argmax}_{\theta \in \Omega } \mathcal {L}(\theta ,\phi ^*). \end{aligned}$$

As the differential equations defining $y(\theta )$ rarely have explicit solutions, the likelihood (2) is difficult to evaluate analytically. It is therefore standard to minimize the negative log-likelihood $G(\theta ,\phi ) = - \log \left( \mathcal {L}(y(\theta ),\phi ^*) \right)$ given by

4 $$\begin{aligned} G(\theta ,\phi ) = \displaystyle \sum _{i=1}^d \log \left( \sqrt{2\pi \sigma _i^2 } \right) + \frac{(y_i(\theta )- \phi _i^*)^2}{\sigma _i^2}. \end{aligned}$$

Under the assumption that $\sigma _i = \sigma$ is fixed, the error term $\log \left( \sqrt{2\pi \sigma ^2 } \right)$ and denominator of $G(\theta ,\phi )$ are constant and do not influence the solution of the optimization problem. The maximum likelihood estimator $\theta ^*$ is the parameter set that minimizes $G(\theta ,\phi ^*)$. A number of computational techniques exist to minimize $G(\theta ,\phi )$ and thus calculate $\theta ^*$. These optimization techniques typically require simulating the mathematical model (1) at each optimization step. Further complicating the optimization problem, $G(\theta ,\phi ^*)$ is often non-convex with multiple local minima.

### Continuation of Maximal Likelihood Estimator

Model fitting is increasingly performed concurrently with experiments (Luo et al. 2022) or obtained from an evolving real-world scenario, as in epidemic modelling (Sanche et al. 2020). In both of these cases, the calibration data $\phi$ evolves and should not be considered as known and constant. In (4), we explicitly wrote the objective function $G(\theta ,\phi )$ as a function of the model parameters $\theta$ and the experimental data $\phi$. The MLE $\theta ^*$ is an implicit function of the experimental data $\phi$ defined by the solution of (3). We are interested in this implicit function $\theta ^*(\phi )$. Most existing optimization techniques consider the calibration data fixed and omit this dependence.

Here, we develop a continuation type technique to compute the evolution of $\theta ^*(\phi )$ numerically as a function of $\phi$ from an initial solution of the optimization problem. We calculate the evolution of $\theta ^*(\phi )$ as the calibration data varies to generate a curve of potential MLEs in $(\phi ,\theta ^*)$ space by developing a numerical continuation technique.

Numerical continuation methods are specialized numerical methods to compute branches of implicitly defined curves. A standard application of these continuation type techniques in mathematical biology is numerical bifurcation analysis (Dhooge et al. 2008; Sanche et al. 2022). These numerical bifurcation techniques compute equilibrium systems of a non-linear dynamical system as a function of model parameters but can be used to detect much richer dynamical behaviour (De Souza and Humphries 2019).

Often, continuation techniques leverage “predictor-corrector” algorithms. Predictor-corrector algorithms predict the solution to a non-linear system of equations using the implicit function theorem (IFT). The IFT is a crucial tool in numerical continuation as it maps a continuation condition to an implicitly defined multivariable function. The IFT states

#### Theorem 1

(Implicit function theorem) Let $F: \mathbb {R}^m \times \mathbb {R}^n \rightarrow \mathbb {R}^m$ be a continuously differentiable function. Assume that

$$\begin{aligned} F(x_0, y_0) = 0 \end{aligned}$$

where $x_0 \in \mathbb {R}^m$ and $y_0 \in \mathbb {R}^n$ and

$$\begin{aligned} \textrm{det}\left( \textrm{D}_x F(x_0,y_0) \right) \ne 0 \end{aligned}$$

where $\textrm{D}_x F(x_0,y_0)$ is the $m\times m$ Jacobian matrix obtained by taking partial derivatives of *F* with respect to *x* at the point $(x_0,y_0)$.

Then, there exists an open set $S \subset \mathbb {R}^n$ with $y_0 \in S$ and a curve *g*(*y*) such that $F(g(y),y) = 0$ for all $y \in S$. Furthermore, *g*(*y*) is continuously differentiable with

5 $$\begin{aligned} \textrm{D}_y g(y) = -[\textrm{D}_x F]^{-1} \textrm{D}_y F. \end{aligned}$$

To illustrate how the IFT facilitates numerical continuation, assume that $F(x_0,y_0)$ satisfies the hypothesis of the theorem. Let *y* be the continuation parameter and we search for solutions of the continuation equation, $F(x,y) = 0$, for *y* in a neighborhood of $y_0$. The IFT ensures that, for a small perturbation $y_1 = y_0 + \Delta y$ of the continuation parameter, there exists a function *g*(*y*) such that $F(g(y_1),y_1) = 0$. Calculating $g(y_1)$ comprises the continuation step of numerical continuation techniques (Meijer et al. 2012). In practice, the continuation step uses the initial solution $x_0 = g(y_0)$ to predict $\hat{x}_1 = g(y_0) + \xi \Delta y$ where $\xi$ is tangent to the solution curve *g*(*y*), although more complex approaches are possible (Meijer et al. 2012; Dhooge et al. 2008). The prediction, $\hat{x}_1$, is then used as a starting point to calculate $g(y_1)$ using standard root finding techniques during the correction step (Meijer et al. 2012).

Here, we develop a “prediction-correction” strategy to predict the behaviour of the solution $\theta ^*(\phi )$ of the inverse problem (3) as a function of the data $\phi$. We focus on the “predictor” step, as the corrector step, if necessary, can utilize existing numerical optimization techniques to calculate the MLE from the predicted MLE.

One of the major steps in developing a continuation method is properly defining the continuation equation. We recall that we are concerned with predicting the evolution of the MLE, which is defined as the minimizer of the log-likelihood. As the log-likelihood (4) is continuously differentiable, local optimal must satisfy

6 $$\begin{aligned} \textrm{D}_\theta G(\theta ^*,\phi ) = 0, \end{aligned}$$

so we necessarily have

$$\begin{aligned} \theta ^*(\phi ) \in \{ \theta \in \Omega | \textrm{D}_{\theta } G(\theta ^*,\phi ) = 0 \}. \end{aligned}$$

However, the optimality condition (6) is a necessary, but not sufficient, condition for $\theta ^*$ to be a MLE. Models that are not structurally identifiable (Raue et al. 2014) have manifolds in parameter space on which this optimality constraint holds but are not necessarily MLEs. We discuss the relationship between our approach and profile likelihood classifications of structural identifiability in Appendix A.

Now, let $\theta ^*_0$ be the MLE for calibration data $\phi _0$. Further, let the Hessian $\textrm{D}^2_\theta G(\theta ,\phi )$ be invertible at $(\theta _0^*,\phi _0) \in \mathbb {R}^p \times \mathbb {R}^d$ and consider the function

$$\begin{aligned} \textrm{D}_{\theta } G(\theta ^*,\phi ): \mathbb {R}^p \times \mathbb {R}^d \rightarrow \mathbb {R}^p. \end{aligned}$$

We take (6) as the continuation equation. As $\theta ^*_0$ is the MLE corresponding to the calibration data $\phi _0$, (6) necessarily holds at $(\theta ^*_0,\phi _0)$. As we have assumed that the Hessian $\textrm{D}^2_\theta G(\theta ,\phi )$ is invertible, we can directly apply the IFT to determine a branch of solutions in $(\theta ,\phi )$ space of (6). The IFT ensures the existence of a function $\Psi (\phi )$ in a neighbourhood of $\phi _0$ with $\Psi (\phi _0) = \theta ^*(\phi _0)$ such that

$$\begin{aligned} \textrm{D}_{\theta } G(\Psi (\phi ) ,\phi ) = 0. \end{aligned}$$

It is natural to consider $\Psi (\phi )$ as the predicted MLE $\theta ^*(\phi )$ for $\phi$ in a neighbourhood of $\phi _0$. However, computing $\Psi (\phi )$ analytically is functionally impossible. We therefore expand $\Psi (\phi )$ as a function of the calibration data $\phi$ using Taylor series

7 $$\begin{aligned} \Psi (\phi +\Delta \phi ) = \Psi (\phi ) + \textrm{D}\Psi (\phi )\Delta \phi + \mathcal {O}(\Delta \phi ^2). \end{aligned}$$

where $\phi + \Delta \phi$ is the updated calibration data and the IFT ensures that the function $\Psi (\phi )$ is continuously differentiable. We calculate $\textrm{D}\Psi (\phi _0)$ to predict $\Psi$ starting from the known solution $\Psi (\phi _0) = \theta _0^*$.

We use the explicit expression given in (5) to calculate $\textrm{D}\Psi (\phi _0)$. In the notation of the IFT, $F(\theta _0,\phi _0) = \textrm{D}_{\theta } G(\theta _0,\phi _0),$ so $F_{\theta } = \textrm{D}^2_{\theta } G(\theta _0,\phi _0)$ and $F_{\phi } = \textrm{D}^2_{\theta ,\phi } G(\theta _0,\phi _0)$. Then, (5) directly implies that

8 $$\begin{aligned} \textrm{D}\Psi (\phi ) = - \left[ \textrm{D}_{\theta }^2 G(\Psi (\phi ),\phi )\right] ^{-1} \textrm{D}_{\theta ,\phi }^2 G(\Psi (\phi ),\phi ). \end{aligned}$$

We then use $\textrm{D}\Psi$ to evaluate (7) and thus perform the continuation step to approximate $\Psi (\phi +\Delta \phi )$.

### Numerical Implementation

We now show how to calculate finite difference approximations to the derivatives included in (8). For $\theta _n$ denoting the *n*-th parameter, we calculate

$$\begin{aligned} \frac{\partial G(\theta ,\phi ) }{\partial \theta _n} = \displaystyle \sum _{i=1}^d 2\left( y_i(\theta ) - \phi _i\right) \frac{ \partial y_i(\theta )}{\partial \theta _n} \end{aligned}$$

and so

9 $$\begin{aligned} \left[ \textrm{D}_{\theta ,\phi }^2 G(\Psi (\phi ),\phi )\right] _{(n,i)} = -2 \frac{ \partial y_i(\theta )}{\partial \theta _n}. \end{aligned}$$

The derivatives $\partial _{\theta _n} y_i(\theta )$ can be calculated through finite difference schemes (Zi 2011)

$$\begin{aligned} \frac{ \partial y_i(\theta )}{\partial \theta _n} = \frac{y_i(\theta +\Delta \theta _n) - y_i(\theta -\Delta \theta _n) }{2\Delta \theta _n} + \mathcal {O}\left( (\Delta \theta _n)^2 \right) , \end{aligned}$$

where $\Delta \theta _n$ is a small perturbation in the *n*-th parameter. Computing $\textrm{D}_{\theta ,\phi }^2 G(\Psi (\phi ),\phi )$ requires 2*p* model simulations for *p* model parameters. We note that $\partial _{\theta _n} y_i(\theta )$ is commonly used to perform local sensitivity analysis (Li and Vu 2013) and that more accurate finite difference approximations, such as centered differences, can be used to calculate $\textrm{D}_{\theta ,\phi }^2 G(\Psi (\phi ),\phi )$.

Calculating the Hessian $\textrm{D}_{\theta }^2 G(\theta ,\phi )$ via finite differences is simple to implement but computationally expensive due to the number of objective function evaluations. In the following examples, we use a finite difference scheme to calculate $\textrm{D}_{\theta }^2 G(\theta ,\phi )$. We calculate the diagonal elements of $\textrm{D}_{\theta }^2 G(\theta ,\phi )$ using forward second order differences and the off-diagonal terms by

$$\begin{aligned} \frac{\partial G(\theta ,\phi )}{\partial \theta _i \partial \theta _j}&= \left( \frac{1}{4(\Delta \theta _i) (\Delta \theta _j)} \right) \left[ G(\theta +\Delta \theta _i+\Delta \theta _j,\phi ) - G(\theta +\Delta \theta _i - \Delta \theta _j,\phi ) \right. \\&{} \quad \left. + \,G(\theta - \Delta \theta _i+\Delta \theta _j,\phi )+ G(\theta -\Delta \theta _i-\Delta \theta _j,\phi ) \right] + \mathcal {O}\left( (\Delta \theta _i)^2, (\Delta \theta _j)^2\right) . \end{aligned}$$

Our computation of the Hessian requires $2p(p+1)$ objective function evaluations.

We note that the Hessian, or the observed Fisher Information, is commonly used throughout parameter optimization algorithms. The Hessian is also used in other techniques such as profile likelihood calculations, estimates of the likelihood function, and classical sensitivity anaylsis. Consequently, computationally efficient techniques to calculate $\textrm{D}_{\theta }^2 G(\theta ,\phi )$ have recently been developed (Stapor et al. 2018).

In fact, many gradient-based optimization techniques approximate the Hessian $D^2_{\theta ,\theta } G(\theta ,\phi )$ at each iteration of solving an optimization problem (MATLAB 2017). For example, both fmincon and fminunc in MATLAB (2017) calculate $D^2_{\theta ,\theta } G(\theta ,\phi )$ at each optimization step and print the pre-computed Hessian as an output of the optimizer. It is therefore possible, and efficient, to recycle this calculation when calculating an update to $\theta _0^*$ using (8).

In total, this numerical implementation of (8) requires $2p(p+2)$ model simulations. Finally, when evaluating (8), it is computationally more appropriate to solve the linear system of equations

$$\begin{aligned} \textrm{D}_{\theta }^2 G(\theta ,\phi ) \textrm{D}\Psi = - \textrm{D}_{\theta ,\phi }^2 G(\Psi (\phi ),\phi ) \end{aligned}$$

for the unknown $\textrm{D}\Psi$.

Code to implement this continuation technique is available at https://github.com/ttcassid/MLE_Continuation.

## Results

### Informing Experimental Design Through the Continuation Method

There are a number of existing techniques to study the relationship between model parameters and data. While our continuation technique focuses on the relationship between the MLE and the calibration data, it has many ties to other existing techniques. Here, we focus on using the explicit relationship between data and the MLE to suggest additional experimental measurements and thus leveraging the continuation method for experimental design. In Appendix A, we discuss how the continuation method relates to parameter identifiability as assessed by the profile likelihood and local sensitivity analysis.

In our derivation of $\textrm{D}\Psi$, we assumed that the Hessian matrix $\textrm{D}_{\theta }^2 G(\theta ,\phi )$ was invertible. The Hessian gives the curvature of the loglikelihood at the MLE and is known as the observed Fisher information matrix $\mathcal {I}_{obs}$. The observed Fisher information is a local measurement in data space. Conversely, the expected Fisher information considers the entirety of data space for fixed model parameters $\theta$. The expected Fisher information is obtained by taking the expectation of $\textrm{D}_{\theta }^2 G(\theta ,\phi )$ over all possible experimental measurements $\phi$ and is defined via

$$\begin{aligned} \mathcal {I} = \mathbb {E}\left[ \textrm{D}_{\theta }^2 G(\theta ,\phi )\right] . \end{aligned}$$

Many existing experimental design methods leverage the expected Fisher information matrix to minimize the covariance in model parameter estimates via the Cramér-Rao inequality. These experimental design techniques typically maximize some aspect, often the determinant, of the Fisher information matrix as a function of possible data to select the most informative calibration data set (Kreutz and Timmer 2009). From a geometric perspective, maximizing the determinant of the Fisher information matrix corresponds to minimizing the volume of the confidence ellipsoid engendered from the covariance matrix (Braniff et al. 2019).

In particular, Braniff et al. (2019) considered the case of bistable gene regulatory networks where the fold bifurcation and unstable manifold between stable equilibria complicates experimental design and parameter estimation. Sharp et al. (2022) considered an information-geometry perspective to propose the expected Fisher information matrix and resulting Riemannian manifold as a guide for data collection. As is often the case, both Sharp et al. (2022) and Braniff et al. (2019) used the expected Fisher information, which considers all possible calibration data via the expectation over $\phi$. Here, we show how our approach complements the classical Fisher information approach to experimental design, albeit through a local measurement, in $(\theta ,\phi )$ space. We recall that

$$\begin{aligned} \textrm{D}\Psi \Delta \phi = -\left[ \mathcal {I}_{obs}\right] ^{-1} \textrm{D}_{\theta ,\phi }^2 G(\Psi (\phi ),\phi ) \Delta \phi . \end{aligned}$$

Now, if $\textrm{D}_{\theta ,\phi }^2 G(\Psi (\phi ),\phi )$ were the identity, then $\textrm{D}\Psi$ would correspond to the observed Fisher information approach to measuring uncertainty in MLE.

In the calculation of $\textrm{D}\Psi \Delta \phi$, the matrix $\textrm{D}_{\theta ,\phi }^2 G(\Psi (\phi ),\phi )$ maps perturbations in the calibration data $\Delta \phi$ through the curvature of the loglikelihood to changes in the MLE. Consequently, $\textrm{D}_{\theta ,\phi }^2 G(\Psi (\phi ),\phi )$ acts as a change of basis matrix from the space of calibration data to parameter space. Simply, $\textrm{D}_{\theta ,\phi }^2 G(\Psi (\phi ),\phi )\Delta \phi$ scales changes in the calibration data to the confidence ellipsoid in parameter space obtained from $\left[ \mathcal {I}_{obs}\right] ^{-1}$. Geometrically, if $\textrm{D}^2_{\theta } G$ has eigenvalues $\lambda _i$ with corresponding eigenvectors $\nu _i$, then choosing $\Delta \phi$ such that

$$\begin{aligned} \nu _i = \textrm{D}^2_{\theta ,\phi } G\Delta \phi \end{aligned}$$

translates perturbations in calibration data to the corresponding eigenspace of the covariance matrix.

For example, the *i*-th column of $\textrm{D}\Psi$ maps perturbations of the *i*-th data point to changes in the MLE. Specifically, the sum

$$\begin{aligned} \frac{\Delta \theta ^*}{\Delta \phi _k} = \displaystyle \sum _{k=1}^p | \textrm{D}\Psi _{k,j} | \end{aligned}$$

measures the sensitivity of the MLE $\theta ^*$ to perturbations in the *k*-th data point. Thus,

$$\begin{aligned} \Vert \textrm{D}\Psi \Vert _{1} = \displaystyle \max _{k= 1,2,...,p} \frac{\Delta \theta ^*}{\Delta \phi _k} \end{aligned}$$

and the most informative data point satisfies

$$\begin{aligned} l = \displaystyle \textrm{argmax}_{k= 1,2,...,p} \displaystyle \frac{\Delta \theta ^*}{\Delta \phi _k} , \end{aligned}$$

where informative is understood as the data point inducing the largest sensitivity in the MLE. As an extreme example, if

$$\begin{aligned} \frac{\Delta \theta ^*}{\Delta \phi _n} = 0, \end{aligned}$$

then perturbations in $\phi _n$ do not impact the MLE estimate, which implies complete insensitivity of the model fit to $\phi _n$. This example corresponds to $\Delta \phi$ belonging to the kernel of the matrix $\textrm{D}^2_{\theta ,\phi }G$ since we have assumed that $\textrm{D}^2_{\theta }G$ is invertible.

We can therefore utilize our analysis to identify which additional experimental measurements could increase confidence in model parameterization. Consider *k* additional measurements $\{\phi _{s,i} = y_{s,i}(\theta ^*)\}_{i=1}^k$ taken directly from the model simulation at times $\{t_{s,i}\}_{i=1}^k$ where the subscript *s* indicates simulated data. Including $\{\phi _{s,i}\}$ in the objective function (4) does not change the MLE or objective value function as these simulated data exactly match the model values. However, $\Vert \textrm{D}\Psi (\phi + \Delta \phi _{s,i})\Vert$ quantifies the sensitivity of the MLE to variability in the *k* simulated measurements. Accordingly, the measurement that maximizes $\Vert \textrm{D}\Psi (\phi + \Delta \phi _{s,i})\Vert$ for a fixed perturbation size $\Delta$ is a good candidate for an additional experimental measurement to decrease parameter uncertainty.

### Examples

The continuation framework derived earlier is applicable to a large variety of models throughout in the mathematical biology literature. To demonstrate the utility of the continuation method, we consider two examples from distinct fields and model formulations. Further, we show how the continuation framework can be leveraged to evaluate the robustness of model parameterizations or identify additional experimental measurements.

First, we consider a classical model of HIV-1 viral dynamics. This model has been used extensively to understand viral dynamics data (Perelson 2002) and the identifiability of model parameters was considered by Wu et al. (2008). In that work, Wu et al. (2008) used simulated data to validate their identifiability results; we follow Wu et al. (2008) and use simulated data to illustrate our approach to predicting the MLE to updated calibration data. We also show how the expression for $\textrm{D}\Psi$ developed for our continuation method can be used to evaluate the robustness of model parameterizations. Quantifying the robustness of model parameterizations is particularly important for practically unidentifiable models, such as the viral dynamics model considered here.

Next, we consider a mathematical model of phenotypic heterogeneity in non-small cell lung cancer (NSCLC) (Cassidy et al. 2021). This model is given by a system of two non-local, structured PDEs representing the density of drug-sensitive and drug-tolerant NSCLC cells. The PDE model is equivalent to a system of ODEs (see (Cassidy et al. 2021) and Appendix C for details). The parameters of the model were fit to in vitro NSCLC data taken from growth experiments in treated and untreated media (Craig et al. 2019; Cassidy et al. 2021). We use this example to demonstrate the effectiveness and computational efficiency of our method to predict the MLE. In addition, we also use the continuation method to suggest additional experimental measurements to increase confidence in model parameterization.

#### Parameter continuation in a viral dynamics model

The standard viral dynamics model has been extensively used to understand the dynamics of viral infection in HIV-1 (Perelson 2002; Hill et al. 2018). The model tracks the concentration of uninfected target cells, *T*(*t*), infected cells *I*(*t*), and free infectious virus *V*(*t*). Here, we follow Wu et al. (2008) and consider a model of HIV-1 dynamics where the target cells are CD$4^+$ T-cells. These cells are produced at a constant rate $\lambda$ and cleared linearly at rate *d*. Infection occurs at a rate $\beta$ following contact between a target cell and infectious viral particle and infected cells are cleared at rate $\delta$. Upon lysis, infected cells release *N* viral particles into the circulation and free virus is cleared at a constant rate *c*. The viral dynamics model is given by

10 $$\begin{aligned} \left. \begin{aligned} \frac{\textrm{d}}{\textrm{dt}}T(t)&= \lambda -\beta T(t) V(t) -d T(t) \\ \frac{\textrm{d}}{\textrm{dt}}I(t)&= \beta T(t) V(t) -\delta I(t) \\ \frac{\textrm{d}}{\textrm{dt}}V(t)&= \delta N I(t) - c V(t). \end{aligned} \right\} \end{aligned}$$

It is common to set $p = \delta N$ so the final equation for *V*(*t*) becomes

$$\begin{aligned} \frac{\textrm{d}}{\textrm{dt}}V(t)&= p I(t) - c V(t), \end{aligned}$$

and the system (10) is equipped with initial conditions $$T(0) = T_0, I(0) =I_0, $$ and $$V(0) = V_0$$. In typical clinical studies, temporal data is only collected for circulating free virus so the model output corresponding to the calibration measurements is

$$\begin{aligned} y_i (\theta ) = \log _{10} (V(t_i,\theta )), \end{aligned}$$

where using $log_{10}$ measurements of viral load is standard in HIV studies.

During antiretroviral therapy (ART), the viral load may fall below the limit of detection of standard assays. While there are a number of techniques to account for this censored data, we do not consider data collected during ART, so the objective function is given by the sum of squares error

11 $$\begin{aligned} G_{HIV}(\theta ,\phi ) = \sqrt{ \displaystyle \sum _{i=1}^n \left( \log _{10}(V(t_i,\theta ) - \log _{10}(\phi _i) \right) ^2 }. \end{aligned}$$

Wu et al. (2008) characterized the identifiability of this model using a higher order derivative method. They found that, if the initial conditions of the model $T_0,I_0,$ and $V_0$ are known, then all six model parameters $\theta = \{ \beta , d, \delta ,c, N, \lambda ,\}$ are identifiable. To illustrate their results, they fixed $\theta = \{ (2 \times 10^{-5}, 0.15, 0.55, 5.5, 900, 80\}$ and simulated the ODE model (10). They sampled the simulated viral load at 37 distinct time points and added noise $\varepsilon _i$ sampled from a Gaussian distribution with $\mu = 0$ and $\sigma ^2=1$ (Wu et al. 2008).

Here, we illustrate how model dynamics evolve during MLE continuation. We follow Wu et al. (2008) but consider a smaller subset of calibration data collected at time $t_i = \{ 0.4, 1, 8, 14, 20, 36, 46, 58\}$. We add noise $\varepsilon _i^0$ sampled from a Gaussian distribution with $\mu = 0$ and $\sigma ^2 = 0.15$ so the initial calibration data is

$$\begin{aligned} \phi _i^0 = \log _{10} (V(t_i,\theta )) + \varepsilon _i^0. \end{aligned}$$

We first fit the model to the simulated data $\phi _i^0$ to obtain an initial MLE. We then generate 4 additional viral load time courses $\{ \phi _i^j\}_{j=1}^{4}$ by

$$\begin{aligned} \phi _i^j = \phi _i^{0} + h_{step} |\varepsilon _i^{j}| \end{aligned}$$

for $\varepsilon _i^j$ sampled from a Gaussian distribution with $\mu = 0$ and $\sigma ^2 = 1$ and $h_{step} = \pm 0.1, \pm 0.2$. This collection of 4 data sets could feasibly represent experimental data measured from an increasingly large sample drawn from a population of HIV-1 positive individuals with population viral dynamic parameters given by $\theta = \{ 2 \times 10^{-5}, 0.15, 0.55, 5.5, 900, 80\}$. Here, we test the ability of our continuation technique to predict reasonable viral dynamic curves *without* refitting the data.

**Fig. 1:**
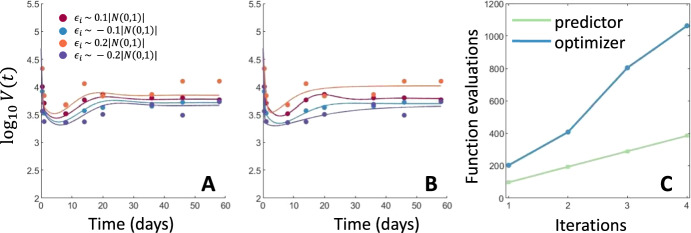
Comparison of predicted model fits to randomly perturbed data. **A** and **B** show model trajectories obtained using predicted and fit model parameters to the simulated experimental data perturbed by $\phi _i^{j} = \phi _i^{0} + h_{step} |\varepsilon _i^{j}|$. Panel **A** shows the predicted model fits to the experimental data while **B** shows the model fits to data resulting from the true MLE. **C** shows the number of objective value evaluations required to predict the MLE using this continuation technique or fit the model parameters to the perturbed data using the known parameters as a starting guess

In Fig. 1A), we compute the predicted $\Psi (\phi ^j)$ and plot the predicted model dynamics obtained from $\Psi (\phi ^j)$ alongside the perturbed data $\phi ^j$ for comparison. In Fig. 1B), we show the fit model predictions, corresponding to the calculated MLE, to the perturbed data. In each case, the viral dynamics show comparable model predictions for the fit and predicted model parameters demonstrating that our continuation method can successfully predict reasonable model simulations. In fact, the Bayesian Information Criteria (Kass and Raftery 1995) indicates no significant differences between the predicted and true MLE for all 4 data sets, which, as we are comparing fits of the same model, corresponds to no significant difference in the objective value function between the predicted and true fits. However, Fig. 1C) shows the significant computational improvement obtained by only calculating the continuation step rather than fitting all model parameters at each step. The predicted model dynamics track the true viral load trajectory.

**Fig. 2:**
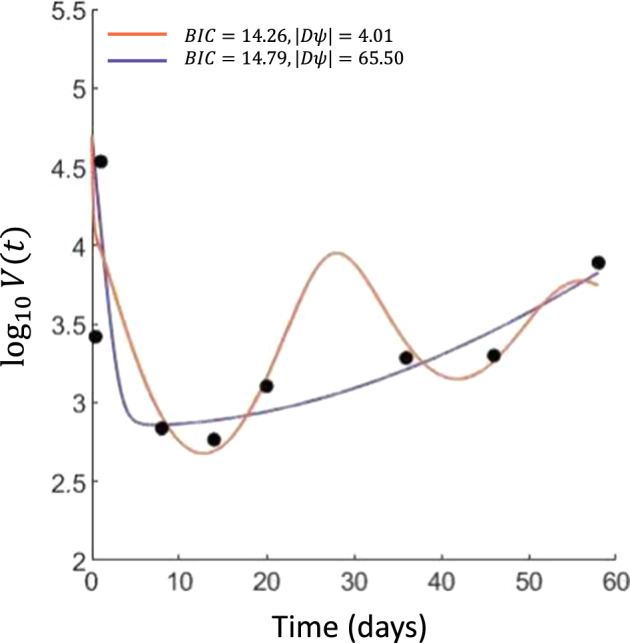
Comparison of two potential fits to randomly perturbed viral dynamics models. Model trajectories obtained from two local minima from fitting 10 initial guesses to viral load data shown in black. Both trajectories accurately describe the viral load dynamics as evidenced by a small difference in BIC. However, the parameter estimate corresponding to the oscillatory trajectory is much more robust as measured by $\Vert \textrm{D}\Psi \Vert$ and is closer to the true underlying parameter set

It is common to find numerous local minima of (11) when fitting (10) to simulated data as the model is practically unidentifiable without precisely knowing the initial conditions. As measured by the value of the log-likelihood function or information criteria, these local minima can produce comparable fits to a given data set despite different dynamics. We perturbed the initial data set $\phi _0$ by

$$\begin{aligned} \log ( \phi _i^1) = \log (\phi _i^{0}) + 0.8 \varepsilon _i \end{aligned}$$

for $\varepsilon _i$ sampled from a Gaussian distribution with $\mu = 0$ and $\sigma ^2 = 1$. We fit this perturbed data from 10 distinct initial guesses using fmincon (MATLAB 2017). These 10 starting initial guesses converged to two local minima. We denote the corresponding parameter estimates by $\hat{\theta }_1$ and $\hat{\theta }_2$ and plot the resulting model trajectories in Fig. 2. These fits both accurately describe the viral load data and are indistinguishable by BIC. As we are comparing fits of the same model, the BIC corresponds to similar objective function values $G(\hat{\theta }_1,\phi )$ and $G(\hat{\theta }_2,\phi )$. Consequently, it is not obvious which of $\hat{\theta }_1$ and $\hat{\theta }_2$ best describe the data.

However, it is reasonable to expect that the MLE should be robust to small perturbations of the calibration data. We measure the robustness of each of these minima by calculating $\Vert \textrm{D}\Psi ( \phi ^1)\Vert$ at $\hat{\theta }_1$ and $\hat{\theta }_2$. While calculating $\Vert \textrm{D}\Psi ( \phi ^1)\Vert$ is not, strictly speaking, a continuation step, we note that

$$\begin{aligned} \textrm{D}\Psi \Delta \phi = \Psi (\phi + \Delta \phi ) - \Psi (\phi ) + \mathcal {O}\left( \Delta \phi ^2 \right) . \end{aligned}$$

Consequently, $\Vert \textrm{D}\Psi \Vert$ measures how robust the potential MLEs $\theta _i$ are to perturbations in the calibration data. As robustness to small perturbations in calibration data is a desirable attribute of the MLE, the continuation framework developed here can evaluate the robustness of potential MLEs, particularly in practically unidentifiable models.

Specifically, a smaller value of $\Vert \textrm{D}\Psi \Vert$ implies less sensitivity of the MLE to perturbations of the calibration data. For the example shown in Fig. 2, there is a 16 fold difference in sensitivity to calibration data. The oscillatory behavior present in Fig. 2 is somewhat surprising as there is no clear indication of oscilliations in the viral load data. However, the sparsity of sampling does not allow for either simulation to be excluded based on the current calibration data alone. Nevertheless, $\textrm{D}\Psi$ can be used to distinguish between these otherwise similar fits. We suggest that, when choosing between multiple fits with similar BIC values, the parameter estimate with the smaller sensitivity to the data is a more robust, and thus preferential, fit.

#### A PDE model of phenotypic switching in mathematical oncology

Non-genetic phenotypic heterogeneity has been increasingly studied as a driver of treatment resistance in solid cancers (Goldman et al. 2015) and a number of mathematical models have recently been developed (Gunnarsson et al. 2020; Jolly et al. 2018; Sahoo et al. 2021; Craig et al. 2019). We consider the Cassidy et al. (2021) model that tracks the density of NSCLC cells with a drug-sensitive (*A*(*t*, *a*)) or drug-tolerant (*B*(*t*, *a*)) phenotype at time *t* and age *a* through an age structured PDE. As mentioned, this PDE model can be reduced to the system of ODEs given in Appendix C. However, we present the simpler biological interpretation of the PDE model here. The total number of cells of each phenotype is given by

12 $$\begin{aligned} {\bar{A}}(t) = \int _0^{\infty } A(t,a)\textrm{d}a \quad \textrm{and} \quad {\bar{B}}(t) = \int _0^{\infty } B(t,a) \textrm{d}a. \end{aligned}$$

The total number of NSCLC cells is given by $N(t) = {\bar{A}}(t) + {\bar{B}}(t)$. Cassidy et al. (2021) considered logistic growth with an Allee effect, wherein cooperation between cells of the same phenotype can lead to increased growth rates, given by

13 $$\begin{aligned} \nonumber R_A({\bar{A}}(t),{\bar{B}}(t))&= r_A\left( 1-\frac{{\bar{A}}(t)+{\bar{B}}(t)}{K} \right) \quad \textrm{and} \\ R_B({\bar{A}}(t),{\bar{B}}(t))&= r_B \left( 1-\frac{{\bar{A}}(t)+{\bar{B}}(t)}{K} \right) f_n({\bar{A}}(t),{\bar{B}}(t)). \end{aligned}$$

where $r_A$ and $r_B$ are phenotype specific growth rates, the carrying capacity is *K*, and the strength of the Allee effect is $f_n({\bar{A}}(t),{\bar{B}}(t)).$ We give the full details of $f_n({\bar{A}}(t),{\bar{B}}(t))$ in Appendix C. Drug-tolerant cells have a constant death rate $d_B$ while the death rate of drug-sensitive cells depends on the presence of anti-cancer treatment via

$$\begin{aligned} d_A = \left\{ \begin{array}{cc} d_A &{} \mathrm {If~untreated,} \\ d_A^{max} &{} \mathrm {during~treatment.} \end{array} \right. \end{aligned}$$

*A*(*t*, *a*) and *B*(*t*, *a*) satisfy the age structured PDEs

14 $$\begin{aligned} \left. \begin{aligned} \partial _t A(t,a) + \partial _a A(t,a)&= -[d_A+R_A({\bar{A}}(t),{\bar{B}}(t))]A(t,a) \\ \partial _t B(t,a) + \partial _a B(t,a)&= -[d_B+R_B({\bar{A}}(t),{\bar{B}}(t))]B(t,a). \end{aligned} \right\} \end{aligned}$$

We detail the corresponding boundary and initial conditions in Appendix C.

The model (14) was fit to in vitro experimental data collected in Craig et al. (2019) using the equivalent ODE formulation for ${\bar{A}}$ and ${\bar{B}}$. In Craig et al. (2019), NSCLC cell population growth was measured in untreated and treated environments. Anti-cancer drugs are applied from day 3 onwards during the treated experiment.

The model is fit to six total calibration data points taken from both the untreated, or control experiment, and the treated experiment. These six measurements correspond to 4 measurements taken at time $t_i = 0,2,4,6$ days during the control experiment, denoted by $\{ \phi _i \}_{i=1}^4$, and 2 measurements taken at time $t_i = 4,6$ days during the treated experiment, denoted by $\{ \phi _i \}_{i=5}^6$. We note that $\phi _3$ and $\phi _5$ were both collected on day 4 of the control and treated experiments, respectively, while $\phi _4$ and $\phi _6$ were both collected on day 6 of the control and treated experiments, respectively. As anti-cancer treatment is applied from day 3 on-wards of the treated experiment and decreases the cancer cell population, we necessarily have $\phi _5 \leqslant \phi _3$ and $\phi _6 \leqslant \phi _4$. We denote the experimental data used to parameterize the model by $\{ \phi _i^0\}_{i=1}^6$. The model output corresponding to the experimental measurements is thus

$$\begin{aligned} y_i (\theta ) = N(t_i,\theta ), \end{aligned}$$

and the objective function is the standard sum of squares error given by

$$\begin{aligned} G_{pheno}(\theta ,\phi ) = \sqrt{ \displaystyle \sum _{i=1}^6 \left( \log _{10}(N(t_i,\theta ) - \log _{10}(\phi _i) \right) ^2 }. \end{aligned}$$

Cassidy et al. (2021) fit model parameters $[r_A, r_B, d_A=d_B, d_A^{max}]$ to treated and untreated experimental data simultaneously for a number of cell lines. The MLE found by Cassidy et al. (2021) corresponds to $\theta ^*(\phi ^0) = [0.4827, 0.3498, 0.7025, 0.4198]$.

We perturbed the experimental data collected by Craig et al. (2019) with increasing amounts of Gaussian noise. We created 10 perturbed data sets $\{ \phi _i^j \}_{i=1}^6$ where the index $j = 1,2,...,10,$ denotes the *j*-th perturbed data set and the normally distributed noise with $\mu = 0$, $\sigma ^2 = 1$, and scaled such that

$$\begin{aligned} \Vert \log _{10}(\phi _i^j) - \log _{10}(\phi _i^*) \Vert = \left( 0.05 + jh_{step} \right) \Vert \log _{10}( \phi _i^0 ) \Vert \end{aligned}$$

where $$ h_{step} = 0.65/55$$ was chosen such that $$\Vert \log _{10}(\phi _i^{10}) - \log _{10}(\phi _i^0) \Vert = 0.75 \Vert \log _{10}( \phi _i^0) \Vert .$$

We enforce that this randomly perturbed data satisfies $\phi _5 \leqslant \phi _3$ and $\phi _6 \leqslant \phi _4$. For each perturbed data set $\{ \phi _i^j \}$, we use the continuation method to calculate

15 $$\begin{aligned} \Psi (\phi ^j) = \theta ^*(\phi ^{j-1} ) + \textrm{D}\Psi (\phi ^{j-1}) \Delta \phi + \mathcal {O}(\Delta \phi ^2). \end{aligned}$$

**Fig. 3:**
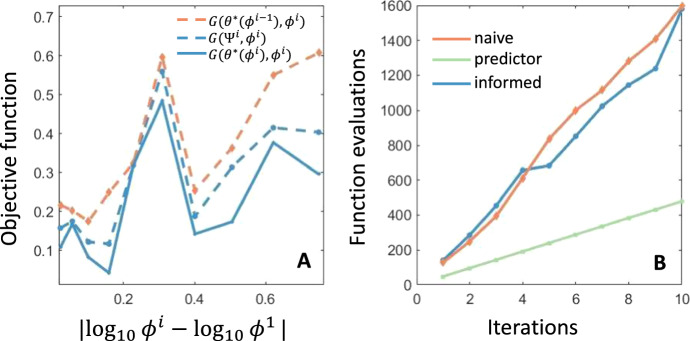
Comparison between MLE estimates obtained using the naive and continuation approaches. **A** shows a comparison of the objective function value for the naive and continuation guesses as well as the true minimal objective function value as a function of the perturbation of the experimental data from the initial data. Here, the naive approach is shown in dashed orange, the predicted approach is shown in dashed blue, and the true minimal objective is in solid blue. **B** shows a comparison of the number of objective value evaluations required to obtain the minimal value when starting from the naive or predicted MLE with the number of function evaluations required to calculate $\Psi (\theta ^i)$

The naive approach to calculate the MLE $\theta ^*(\phi ^j)$ for updated data $\phi ^j$ would be to use the MLE from the previous data, $\theta ^*(\phi ^{j-1})$, as an initial starting guess for the parameter fitting step. Hence, to illustrate the utility of our continuation technique, we calculated $\Psi (\phi ^j)$ using (15) and then calculated $G_{pheno}(\Psi ( \phi ^j ),\phi ^j)$. We also calculated the true MLE $\theta ^*(\phi ^j)$ using the Matlab algorithm fmincon from both starting guesses $\Psi (\phi ^j)$ and $\theta ^*(\phi ^{j-1} )$. In Fig. 3A), we show the objective function value evaluated at the updated data $\phi ^j$ and three parameter sets: the naive starting point, $\theta ^*(\phi ^{j-1})$; the predicted MLE, $\Psi (\phi ^j)$; and the true MLE, $\theta ^*(\phi ^j)$.

The non-monotonic profile of the objective function $G_{pheno}$ in Fig. 3A) is to be expected as we are adding noise to experimental data. This noise may perturb the existing data away from dynamics that can be well-described by the mathematical model. Accordingly, the important information from Fig. 3A) is the comparison

$$\begin{aligned} G_{pheno}(\theta ^*(\phi ^i),\phi ^i) \leqslant G_{pheno}(\Psi (\phi ^i),\phi ^i) < G_{pheno}(\theta ^*(\phi ^{i-1}),\phi ^i), \end{aligned}$$

which demonstrates the accuracy of the continuation step (7) in driving a relative decrease in $G_{pheno}$.

Further, in Fig. 3B), we show the cumulative number of objective function evaluations when calculating $\theta ^*(\phi ^j)$ for $j = 1,2,...,10$ when starting the optimization from $\theta ^*(\phi ^{j-1})$ and $\Psi (\phi ^j)$. The total number of function evaluations used is lower when starting the optimization from the predicted MLE $\Psi (\phi ^j)$ than when starting from $\theta ^*(\phi ^{j-1})$. More strikingly, the predicted MLE $G(\Psi (\phi ^j),\phi ^j)$ is comparable against $G(\theta ^*(\phi ^{j},\phi ^j))$ in Fig. 3A) and there is notable computational benefit to only calculating the predicted MLE $\Psi (\phi ^j)$ rather than re-fitting the parameters. Taken together, the results shown in Fig. 3 demonstrate the accuracy and computation efficiency gained by calculating $\Psi (\phi ^j)$.

We next utilize the continuation framework to identify additional time points to increase confidence in model parameters. We focus on the treated environment and consider additional time points $t_{s,i} = 3.1,3.2,3.3,3.4,3.5,5,7$ days with corresponding simulated measurements $\{ \phi _{i,s} \}_{i = 1}^7 = N(t_{s,i}).$ We perturb each of these simulated measurements by a fixed amount, $\Delta \phi = \pm 0.3 N(3.1)$, to give 14 additional, perturbed measurements. We appended each of these 14 measurements to the experimental data and predicted the MLE to these appended data sets.

We calculated the relative change in the MLE for each model parameter and each of the 14 appended data sets. Each of the simulated data point occurs following the beginning of therapy, although it would be simple to test other additional experimental measurements. The immediate decrease observed in *N*(*t*) following the beginning of treatment is due to the death of sensitive cells following treatment administration and controlled by the parameter $d_A^{max}$. From the biological interpretation of the parameters, we expect $d_A^{max}$ to be highly sensitive to perturbations in these data points.

**Fig. 4:**
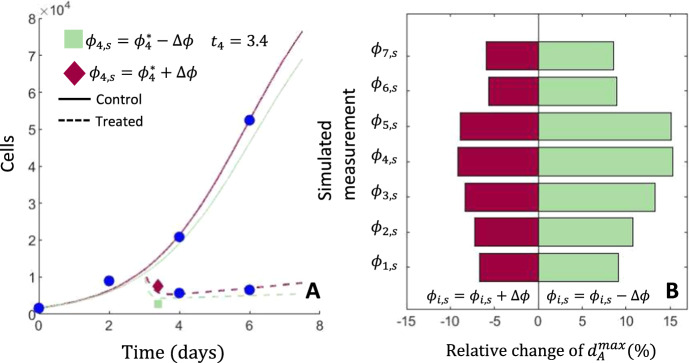
Evaluating additional time points to identify $d_{A}^{max}$ in an in vitro model of NSCLC. **A** shows the a selection of predicted model dynamics when fit to experimental data with a single additional time point $\phi _{i,s}^*$ that is perturbed by a $\Delta \phi$ from the true simulated value. For figure clarity, the model trajectories corresponding to the perturbation of $\{ \phi _{4,s} \}$ are shown. Here, the solid line corresponds to the control experiment while the results of the treated experiment are plotted with a dashed line. **B** shows a tornado plot of the predicted relative change in the best-fit parameter $d_{A}^{max}$ for each additional simulated data point $\{ \phi _{i,s} \}_{i = 1}^7$ where the perturbation $\Delta \phi > 0$. The left side of the tornado plot, in red, shows the relative change when the perturbed value $\phi _{i,s} = \phi _{i,s}^* + \Delta \phi$ is larger than the simulated value $\phi _{i,s}^*$. The right-hand side, in green, shows the relative change in $d_a^{max}$ when $\phi _{i,s} = \phi _{i,s}^* - \Delta \phi$ is smaller than the simulated value $\phi _{i,s}^*$

As expected, $d_A^{max}$ was the most sensitive model parameter to perturbations of the simulated data. We show the percent relative change in $d_a^{max}$ from the unperturbed data in Fig. 4B. We plot the relative change of the other model parameters for the same perturbations in Appendix C. As expected, the maximal death rate of sensitive cells increased when the simulated data point was decreased from the true value and decreased when the simulated data point was increased.

The treatment sensitive population rapidly shrinks during therapy. The stabilization and rebound of the population during therapy is due to the expansion of the drug resistant population. This stabilization occurs once the drug sensitive population has been maximally suppressed which due to the drug effect. The most informative simulated data point, as measured by the magnitude of the relative change in the parameter $d_A^{max}$, was at time $t_{i,s} = 3.4$. At $t = 3.4$, drug sensitive cells are no longer dominant due to drug pressure. The depth of the population response to treatment, as measured by *N*(3.4),  is thus highly sensitive to death rate of these drug sensitive cells under treatment. However, expecting an additional experimental measurement to be made at precisely 3.4 days is unrealistic due to experimental constraints. However, Fig. 4B shows that measurements at $t_{s,3} = 3.3$ and $t_{s,3}= 3.5$ would also strongly inform $d_A^{max}$. Consequently, our conclusion that $t_{s,4}= 3.4$ is the most informative time for an additional experimental measurement is robust to the fact that an experimental measurement cannot be made at precisely 3.4 days. Our results indicate that including an additional experimental measurement in the 4.8 h window between 3.3$-$3.5 days will strongly inform $d_A^{max}$, which is experimentally feasible.

In Fig. 4A, we show the simulated experimental measurements and predicted model dynamics for the most informative time point. The predicted model simulations capture the perturbed data point while retaining good fits to the true experimental data.

## Discussion

Parameter fitting is crucial step when using mathematical models to predict novel treatment strategies, extrapolate from clinical trials, identify new drug targets or schedules, or propose non-pharmaceutical interventions (Brady and Enderling 2019; Cassidy et al. 2020; Cassidy and Craig 2019). However, parameter fitting can be difficult and computationally expensive. A large variety of fitting techniques have therefore been developed to calibrate model predictions against data (Toni et al. 2009; Horbelt et al. 2002; Kreutz et al. 2013; Lauß et al. 2018). Moreover, mathematical modeling is increasingly applied to understand emerging data and make real-time predictions. In this case, as new data emerges, the model parameters must be refit with potential computational cost. Here, we developed a continuation type technique to quantify how updates to experimental data will impact the MLE and predict the evolution of the MLE as a function of the experimental data used to calibrate the model.

We used the IFT to calculate the trajectory of the MLE through parameter space. As the IFT only guarantees the existence of a differentiable trajectory $\Psi$ through calibration data–parameter space, we utilized the first order Taylor expansion $\Psi$ to extrapolate the evolution of the MLE due to changes in experimental data. We showed how this calculation is intrinsically linked to local sensitivity analysis and the curvature of the objective function. In two examples drawn from mathematical biology, we showed how this continuation technique can predict acceptable model fits to experimental data while significantly reducing computational overhead. In fact, in most applications, our continuation technique requires no dedicated computational overhead as the Hessian of the objective function is calculated at each step when using common optimization algorithms, such as fmincon (MATLAB 2017), and local sensitivity analysis is a standard step in model fitting.

Perhaps more importantly that gains in computational efficiency, our approach explicitly identifies relationships between individual experimental measurements and parameter estimates. Our approach addresses similar questions to local sensitivity analysis from a distinct perspective. Rather than using simulations to understand how small perturbations in model parameters from the best-fit parameters change model outputs as in standard sensitivity analysis, we quantify how changes in the training data impact the best-fit parameters and measure the sensitivity of the best-fit parameters to variations in this calibration data. As we showed in Sect. 2, this perspective can be used to suggest additional experimental measurements to increase confidence in model parameterization. Further, we showed how to use $D\Psi$ to understand which experimental measurements are most informative for model parameterizations and identify redundant measurements that do not provide additional information for parameter estimation.

Our technique is a type of local analysis that explores the functional dependence of the MLE on experimental data starting from a pre-identified MLE. Specifically, we assume that the Hessian of the objective function is invertible at the MLE and our results are necessarily local in parameter space as we are extrapolating from a pre-identified MLE. Nevertheless, our examples show the utility of our continuation approach for even large perturbations of the experimental data.

Despite these limitations, we developed a continuation-type technique to predict the functional dependence of a MLE on the experimental data used to train a mathematical model. While we have focused on applications in mathematical biology, our approach is immediately portable to other domains. As our method is independent of the number of data points, our approach could be particularly useful in big-data applications. Ultimately, our results offer a unified approach to quantify the relationship between training data and best-fit model parameters and to leverage this understanding to suggest additional experiments to increase confidence in model parameterization.

## Figures and Tables

**Fig. 5:**
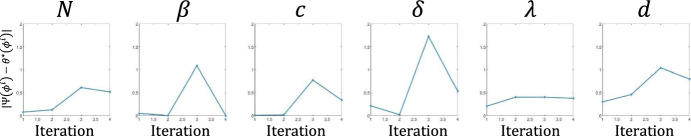
Absolute error between the predicted model parameter obtained using the continuation method and best fit parameter obtained by fitting the data for the viral dynamics shown example in Fig. 1. Each panel shows the absolute error for each model parameter plotted against the iteration number

**Fig. 6:**
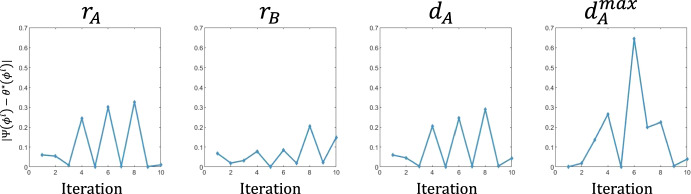
Absolute error between the predicted model parameter obtained using the continuation method and best fit parameter obtained by fitting the data for the phenotype model example shown in Fig. 3. Each panel shows the absolute error for each model parameter plotted against the iteration number

**Fig. 7:**
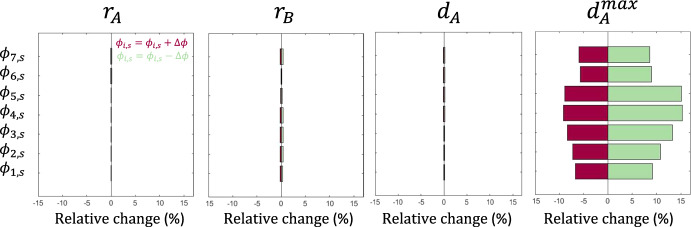
Tornado plots of the predicted relative change in the best-fit parameters for each additional simulated data point. Each panel shows the predicted change to the model parameters $r_a,r_b,d_a$ and $d_a^{max}$ for the perturbed data points $\{ \phi _{i,s} \}_{i = 1}^7$ where the perturbation $\Delta \phi _{i,s} > 0$.In each tornado plot, the red bar shows the relative change in each model parameter when the perturbed value $\phi _{i,s} = \phi _{i,s}^* + \Delta \phi$ is larger than the simulated value $\phi _{i,s}^*$. The green bar shows the relative change in each model parameter when $\phi _{i,s} = \phi _{i,s}^* - \Delta \phi$ is smaller than the simulated value $\phi _{i,s}^*$

## Data Availability

The code and data underlying the results in this manuscript are available at https://github.com/ttcassid/MLE_Continuation.

## Appendix A: Relationships Between MLE Continuation and Exisiting Techniques

### A.1 Parameter Identifiability

In the main text, we have explicitly written the MLE estimator as a function of the experimental data used to fit a model. This approach is intrinsically related to parameter identifability analysis. Identifiability analysis attempts to determine if available experimental observations are capable to uniquely determine model parameters. Accordingly, the practical identifiability of a mathematical model depends on available experimental data. The *profile likelihood*, given by

$$\begin{aligned} PLE_{\theta _i}(c) = \min _{\theta _i = c, \theta \in \mathbb {R}^p } G(\theta ,\phi ), \end{aligned}$$

and introduced by Raue et al. (2009), is a projection of the likelihood function onto the model parameter $\theta _i = c$. The profile likelihood illustrates the behaviour of the likelihood function as the parameter $\theta _i$ is fixed away from the optimal value $\theta ^*_i$. The shape of $PLE_{\theta _i}(c)$ illustrates the confidence interval of the parameter estimate $\theta _i^*$ for given experimental data. Formally, Raue et al. (2009) define these confidence intervals by

$$\begin{aligned} \mathrm {C.I.}(\theta _i,\alpha ) = \{ c | PLE_{\theta _i}(c) - PLE_{\theta _i}(\theta _i^*) < \Delta _{\alpha } \} \end{aligned}$$

where $\Delta _{\alpha } = \chi ^2(\alpha ,df)$ is the $\chi ^2$ distribution at significance level $\alpha$ and *df* degrees of freedom (Raue et al. 2009). A parameter is practically identifiable in the sense of Raue et al. (2009) with confidence level $1-\alpha$ if $\mathrm {C.I.}(\theta _i,\alpha )$ is bounded in parameter space for given experimental data. Conversely, a non-identifiable parameter has a profile likelihood that does not increase past the threshold $\Delta _{\alpha }$.

The profile likelihood is intrinsically linked to the available experimental data $\phi _i$. We view the PLE as a function of both the parameter $\theta _i$ and the experimental data $\phi$

$$\begin{aligned} PLE_{\theta _i}(c,\phi ) = \min _{\theta _i = c, \theta \in \mathbb {R}^p } G(\theta ,\phi ). \end{aligned}$$

For practically unidentifiable models, it is natural to ask what perturbations to the experimental data could render the model practically identifiable. Raue et al. (2009) use the profile likelihood of a model parameter to suggest additional experiments to resolve practical non-identifiability. They simulate the model for parameter values along $PLE_{\theta _i}$ to suggest additional experimental measurements at times $t_{s,i}$, where $t_{s,i}$ represents the *i*-th *simulated* measurement time. In our framework, we define

$$\begin{aligned} \theta ^*|_{\theta _i = c }(\phi ) = \textrm{argmin}_{\theta _i = c, \theta \in \mathbb {R}^p} G(\theta ,\phi ), \end{aligned}$$

so that

$$\begin{aligned} PLE_{\theta _i}(c,\phi ) = G(\theta ^*|_{\theta _i = c }(\phi ) ,\phi ). \end{aligned}$$

We note that the definition of $\theta ^*|_{\theta _i = c }(\phi )$ is precisely that of $\theta ^*(\phi )$ with the added constraint that $\theta _i = c$. We can calculate $\textrm{D}_{\phi } \theta ^*|_{\theta _i = c }$ as a function of the experimental data $\phi$ in precisely the same manner as described previously. Consequently, our continuation approach can complement the experimental design approach suggested by Raue et al. (2009) by incorporating the sensitivity of the MLE to perturbations in the (simulated or experimental) calibration data.

### A.2 Sensitivity analysis

Local sensitivity analysis quantifies how small perturbations of the best-fit parameters impact model output (Zi 2011). A standard approach to local sensitivity analysis is using the finite difference approximation of

$$\begin{aligned} S_n(t_i) = \frac{ \partial y (\theta )}{\partial \theta _n} = \frac{h(t_i,\theta +\Delta \theta _n) - h(t_i,\theta -\Delta \theta _n) }{2\Delta \theta _n} + \mathcal {O}\left( \Delta \theta _n \right) \end{aligned}$$

to identify which parameter values strongly impact model projections. When $|S_n|$ is small, the model output is considered to be insensitive to $\theta _n$. The *n*-th row of $\textrm{D}_{\theta ,\phi }^2 G(\Psi (\phi ),\phi )$ is precisely $S_n(t_i)$ for $t_i$ corresponding to calibration data measurements. When implementing (7), the magnitude of the continuation step $\textrm{D}\Psi (\phi )\Delta \phi$ in the direction of $\theta _n$ is scaled by $S_n$. This scaling encodes the local sensitivity of model predictions to variations in parameters in the prediction of $\Psi (\phi )$. Consequently, our continuation method naturally includes the information gained from local sensitivity analysis.

## Appendix B: Viral Dynamics Model

In Fig. 5, we plot the absolute error between the model parameters obtained by our continuation method alongside the best fit parameters represented in Fig. 5. Here, we fit the natural log of the viral dynamics parameters. The predicted parameter value is identical to the MLE for some, although not all, iterations.

## Appendix C: Structured PDE Model of Tumour Dynamics

Here, we give additional details regarding the phenotype PDE in (14). The proofs of these results and more information can be found in Cassidy et al. (2021).

### C.1 Model Description

In Sect. 2, we considered a structured PDE model of cancer phenotypic plasticity developed in Cassidy et al. (2021). The PDE model tracks the age density of drug sensitive and drug tolerant cells through (14) where the variable *a* in (14) corresponds to chronological cellular age. The left-hand side of (14) is strictly negative, as is typical in age-structured populations models that distinguish between the loss of mother cells due to mitosis and the appearance of daughter cells with age $a =0$ Perthame (2007); Cassidy et al. (2019). Specifically, cellular reproduction is included in the Cassidy et al. (2021) model via the boundary condition for *A*(*t*, 0) and *B*(*t*, 0). Cassidy et al. (2021) assumed that cells can change phenotype at birth and that older mother cells are more likely to produce daughter cells that change phenotype Arora et al. (2017); Uetake and Sluder (2010). Then, the boundary condition of (14) is given by

$$\begin{aligned} \left. \begin{aligned} A(t,0)&= 2 \hspace{-2pt}\int _0^{\infty } \hspace{-10pt} \left[ R_A({\bar{A}}(t),{\bar{B}}(t)) \beta _{AA}(a)A(t,a) \right. \\&\quad \left. {} + R_B({\bar{A}}(t),{\bar{B}}(t))\beta _{BA}(a)B(t,a) \right] \textrm{d}a\\ B(t,0)&= 2 \hspace{-2pt}\int _0^{\infty } \hspace{-10pt} \left[ R_A({\bar{A}}(t),{\bar{B}}(t)) \beta _{AB}(a)A(t,a) \right. \\&\quad {} \left. + R_B({\bar{A}}(t),{\bar{B}}(t))\beta _{BB}(a)B(t,a) \right] \textrm{d}a. \end{aligned} \right\} \end{aligned}$$

where

$$\begin{aligned} \beta _{ii}(a) = P_{ii}^* + (P_{ii}^{max}-P_{ii}^*)\exp \left[ -\sigma _{i} a \right] \end{aligned}$$

and $\beta _{ij}(a) = 1-\beta _{ii}(a)$. These terms give the probability of a parent cell of phenotype *i* and age *a* will produce a daughter cell with phenotype *j*. We refer to Cassidy et al. (2021) for a discussion of the functional forms and parameterization of $\beta _{ij}$.

As mentioned in the Main Text, Cassidy et al. (2021) considered an Allee effect in the growth rate of drug tolerant cells. The function $f_n({\bar{A}}(t),{\bar{B}}(t))$ is given by

$$\begin{aligned} f_n({\bar{A}}(t),{\bar{B}}(t))&= 1 +\left( \frac{r_A-r_B}{r_B}\right) \left( \frac{{\bar{B}}(t)^n}{{\bar{A}}(t)^n+{\bar{B}}(t)^n } \right) \\ \nonumber&= 1 +\left( \frac{r_A-r_B}{r_B}\right) \left( \frac{\theta (t)^n}{1+\theta (t)^n } \right) = f_n(\theta (t)) \quad \textrm{for} \quad \theta (t) = {\bar{B}}(t)/{\bar{A}}(t). \end{aligned}$$

When fitting (14) to data, we are primarily interested in the total number of cells, rather than their age density. Consequently, we focus on ${\bar{A}}(t)$ and ${\bar{B}}(t)$. Cassidy et al. (2021) derived the following system of ODEs for these quantities

16 $$\begin{aligned} \left. \begin{aligned} \frac{\textrm{d}}{\textrm{dt}}{\bar{A}}(t)&= -[R_A({\bar{A}}(t),{\bar{B}}(t)) + d_A ] {\bar{A}}(t) \\&\quad {} + 2R_A({\bar{A}}(t),{\bar{B}}(t)) N_{AA}(t) + 2R_B({\bar{A}}(t),{\bar{B}}(t)) \left[ {\bar{B}}(t) - N_{BB}(t) \right] \\ \frac{\textrm{d}}{\textrm{dt}}{\bar{B}}(t)&= -[R_B({\bar{A}}(t),{\bar{B}}(t)+d_B] {\bar{B}}(t) + 2R_A({\bar{A}}(t),{\bar{B}}(t))\left( {\bar{A}}(t) - N_{AA}(t) \right) \\&{} \quad + 2R_B({\bar{A}}(t),{\bar{B}}(t)) N_{BB}(t) \\ \frac{\textrm{d}}{\textrm{dt}}N_{AA}(t)&= P_{AA}^{max} \left[ 2R_A({\bar{A}}(t),{\bar{B}}(t)) N_{AA}(t) + 2R_B({\bar{A}}(t),{\bar{B}}(t))\left( {\bar{B}}(t)-N_{BB}(t)\right) \right] \\&\quad {} - \left( R_A({\bar{A}}(t),{\bar{B}}(t))+ d_A \right) N_{AA}(t) + \sigma _A \left( P^*_A \bar{A}(t) - N_{AA}(t) \right) \\ \frac{\textrm{d}}{\textrm{dt}}N_{BB}(t)&= P_{BB}^{max} \left[ 2R_A({\bar{A}}(t),{\bar{B}}(t))\left( {\bar{A}}(t)-N_{AA}(t)\right) + 2R_B({\bar{A}}(t),{\bar{B}}(t)) N_{BB}(t) \right] \\&\quad {} - \left( R_B({\bar{A}}(t),{\bar{B}}(t))+d_B \right) N_{BB}(t) -\sigma _B N_{BB}(t) + \sigma _B P^*_B \bar{B}(t). \\ \end{aligned} \right\} \nonumber \\ \end{aligned}$$

We use the system ODEs in (16) to simulate the model when performing MLE continuation. We use the initial conditions derived by Cassidy et al. (2021) to equip the initial value problem. For a population in stable exponential growth with growth rate $\lambda _p$, we have

$$\begin{aligned} {\bar{A}}(0) = \frac{A_0}{r_A+d_A+\lambda _p} \quad \textrm{and} \quad {\bar{B}}(0) = \frac{B_0}{r_B+d_B+\lambda _p} \end{aligned}$$

where $A_0$ and $B_0$ are linked to the initial age distribution of drug-sensitive and drug-tolerant cells. It follows that

$$\begin{aligned} N_{AA}(0)&= \int _0^{\infty } \left( P_{AA}^* + (P_{AA}^{max}-P_{AA}^*)e^{-\sigma _A a} \right) A_0 \exp (-(r_A+d_A+\lambda _p) a ) \textrm{d}a \\&= A_0 \left( \frac{P_{AA}^*}{r_A+d_A+\lambda _p} + \frac{ P_{AA}^{max}-P_{AA}^* }{r_A+d_A+\lambda _p+\sigma _A}\right) , \end{aligned}$$

and

$$\begin{aligned} N_{BB}(0)&= B_0 \left( \frac{P_{BB}^*}{r_B+d_B+\lambda _p} + \frac{ P_{BB}^{max}-P_{BB}^* }{r_B+d_B+\lambda _p+\sigma _B} \right) . \end{aligned}$$

### C.2 Comparison Between Predicted and True MLE

In Fig. 3, we compared the objective value function corresponding to the naive, predicted, and fit MLE. Those results demonstrate that the predicted MLE produces similar model trajectories to the fit MLE. Here, we show a comparison between parameter estimates of the true and predicted MLE in Fig. 6. We note that, for some iterations, the predicted parameter value was identical to the parameter value found through optimization.

### C.3 Experimental Design Effects on Other Model Parameters

As mentioned in the main text, the death rate of drug sensitive cells was the most sensitive parameter to perturbations of the simulated data. However, the other parameters were also predicted to change in response to perturbations of the simulated data. We show the relative changes for the other model parameters at each of the 7 simulated data points $\{ \phi \}_{i=1}^7$ in Fig. 7. Unsurprisingly from the biological interpretation of the parameters, $d_A^{max}$ is the most sensitive to the perturbed simulated data. However, there is a small increase in the growth rate of drug-tolerant cells, $r_B$, when the simulated data is perturbed by a negative amount. This can be understood by a fitness-increase of the drug-tolerant cells and thus stronger competition between the drug-tolerant and drug-sensitive cells. This competition decreases the population size prior to treatment which allows for a better fit to the perturbed data point post-treatment despite a larger resistant population.
